# Global Genomic Analysis of SARS-CoV-2 RNA Dependent RNA Polymerase Evolution and Antiviral Drug Resistance

**DOI:** 10.3390/microorganisms9051094

**Published:** 2021-05-19

**Authors:** Alfredo Mari, Tim Roloff, Madlen Stange, Kirstine K. Søgaard, Erblin Asllanaj, Gerardo Tauriello, Leila Tamara Alexander, Michael Schweitzer, Karoline Leuzinger, Alexander Gensch, Aurélien E. Martinez, Julia Bielicki, Hans Pargger, Martin Siegemund, Christian H. Nickel, Roland Bingisser, Michael Osthoff, Stefano Bassetti, Parham Sendi, Manuel Battegay, Catia Marzolini, Helena M. B. Seth-Smith, Torsten Schwede, Hans H. Hirsch, Adrian Egli

**Affiliations:** 1 Applied Microbiology Research, Department of Biomedicine, University of Basel, 4056 Basel, Switzerland; alfredo.mari@unibas.ch (A.M.); tim.roloff@usb.ch (T.R.); madlen.stange@usb.ch (M.S.); kirstinekobberoee.soegaard@usb.ch (K.K.S.); michael.schweitzer@usb.ch (M.S.); alexander.gensch@unibas.ch (A.G.); Helena.Seth-Smith@usb.ch (H.M.B.S.-S.); 2 Division of Clinical Bacteriology and Mycology, University Hospital Basel, 4031 Basel, Switzerland; 3 SIB Swiss Institute of Bioinformatics, 4053-4056 Basel, Switzerland; erblin.asllanaj@unibas.ch (E.A.); gerardo.tauriello@unibas.ch (G.T.); leila.alexander@unibas.ch (L.T.A.); torsten.schwede@unibas.ch (T.S.); 4 Biozentrum, University of Basel, Klingelbergstrasse 50-70, CH-4056 Basel, Switzerland; 5 Clinical Virology, University Hospital Basel, 4031 Basel, Switzerland; karoline.leuzinger@usb.ch (K.L.); hans.hirsch@usb.ch (H.H.H.); 6 Transplantation & Clinical Virology, Department of Biomedicine, University of Basel, 4031 Basel, Switzerland; 7 Infectious Diseases and Hospital Epidemiology, University Hospital Basel and University of Basel, 4031 Basel, Switzerland; Aurelien.Martinez@usb.ch (A.E.M.); parham.sendi@ifik.unibe.ch (P.S.); manuel.battegay@usb.ch (M.B.); catia.marzolini@usb.ch (C.M.); 8 Pediatric Infectious Diseases, University of Basel Children’s Hospital, 4056 Basel, Switzerland; julia.bielicki@ukbb.ch; 9 Intensive Care Medicine, University Hospital Basel, 4031 Basel, Switzerland; hans.pargger@usb.ch (H.P.); martin.siegemund@usb.ch (M.S.); 10 Emergency Medicine, University Hospital Basel, 4031 Basel, Switzerland; Christian.Nickel@usb.ch (C.H.N.); roland.bingisser@usb.ch (R.B.); 11 Internal Medicine, University Hospital Basel, 4031 Basel, Switzerland; Michael.Osthoff@usb.ch (M.O.); Stefano.Bassetti@usb.ch (S.B.); 12 Institute for Infectious Diseases, University of Bern, 3001 Bern, Switzerland

**Keywords:** diagnostics, surveillance, resistance, evolution, SARS-CoV-2, remdesivir, genome analysis, RNA dependent RNA polymerase

## Abstract

A variety of antiviral treatments for COVID-19 have been investigated, involving many repurposed drugs. Currently, the SARS-CoV-2 RNA-dependent RNA polymerase (RdRp, encoded by *nsp12-nsp7-nsp8*) has been targeted by numerous inhibitors, e.g., remdesivir, the only provisionally approved treatment to-date, although the clinical impact of these interventions remains inconclusive. However, the potential emergence of antiviral resistance poses a threat to the efficacy of any successful therapies on a wide scale. Here, we propose a framework to monitor the emergence of antiviral resistance, and as a proof of concept, we address the interaction between RdRp and remdesivir. We show that SARS-CoV-2 RdRp is under purifying selection, that potential escape mutations are rare in circulating lineages, and that those mutations, where present, do not destabilise RdRp. In more than 56,000 viral genomes from 105 countries from the first pandemic wave, we found negative selective pressure affecting *nsp12* (Tajima’s D = −2.62), with potential antiviral escape mutations in only 0.3% of sequenced genomes. Potential escape mutations included known key residues, such as Nsp12:Val473 and Nsp12:Arg555. Of the potential escape mutations involved globally, in silico structural models found that they were unlikely to be associated with loss of stability in RdRp. No potential escape mutation was found in a local cohort of remdesivir treated patients. Collectively, these findings indicate that RdRp is a suitable drug target, and that remdesivir does not seem to exert high selective pressure. We anticipate our framework to be the starting point of a larger effort for a global monitoring of drug resistance throughout the COVID-19 pandemic.

## 1. Introduction

Infection with SARS-CoV-2 is associated with substantial morbidity and mortality. While no approved therapy is available to-date, there have been multiple recent efforts to repurpose existing drugs. Understanding how the mode of action and efficacy of a potential antiviral relates to selective pressure for the evolution of resistance will be vital in ensuring that effective antivirals remain viable treatment options. In this context, the surveillance for potential emergence of antiviral resistance is paramount for public health during the COVID-19 pandemic. The first drugs considered for antiviral treatment were inhibitors targeting 3C-like proteases (3CL-Pro) and Spike proteins (S) [1,2]. However, the alarming number of side effects and lack of clinical efficacy forced the identification of new targets of viral replication machinery [3]. Owing to its high degree of amino acid conservation within beta-coronaviridae, RNA dependent RNA polymerase (RdRp) (over 96% identity [4]) is a key target of antiviral drug development, and recently, of drug-repurposing [4,5]. Several studies have indicated in vitro efficacy against SARS-CoV-2 of potential inhibitory candidates such as remdesivir, sofosbuvir, galidesivir, tenofovir, and ribavirin [6,7,8]. All of these inhibitors share the same mechanism of action, binding RdRp in its active site as nucleoside analogues and interrupting RNA polymerization [6] (Supplementary Figures S1 and S2A).

Antiviral treatments have been shown to lead to the emergence of resistance in Hepatitis B Virus, Human Immunodeficiency Virus, Hepatitis C Virus (HCV), and influenza virus [9,10,11]. For example, the emergence of ribavirin resistant mutants has been described for HCV RdRp and has been largely attributed to the evolution of resistance-conferring single nucleotide polymorphisms (SNPs), usually resulting in a remarkable fitness loss [10,12]. Similarly, in vitro experiments on SARS-CoV, causative agent of Severe Acute Respiratory Syndrome (SARS), and the closest relative of SARS-CoV-2, show that specific SNPs within *nsp12*, the major RdRp subunit, may alter the effectiveness of remdesivir [13]. The substitution Nsp12:Phe480Leu destabilizes the interface between different sub-domains of the protein (“palm” and “fingers”, Supplementary Figure S1A,B), likely affecting the proof-reading capacity of RdRp [4]. Nsp12:Val557Leu affects binding to the template RNA and indirectly to remdesivir [4] (Supplementary Figure S1C,D). The EC_50_ of remdesivir increased six-fold from 0.01 µM to 0.06 µM in cultures of SARS-CoV carrying Nsp12:Phe480Leu or Nsp12:Val557Leu mutations [13]. In the absence of remdesivir, these viral mutants were found to replicate less efficiently, and showed a substantially reduced fitness [13].

The clinical efficacy of remdesivir in COVID-19 treatment has been recently debated. Two studies suggested a reduction in recovery time [14,15], while others did not show a reduction of mortality [16,17]. At present, expert panels from authorities (Center for Disease Control and Prevention (CDC), National Institute of Health (NIH)) have made recommendations on the use of remdesivir in patients at high risk of disease progression or requiring supplemental oxygen (https://www.covid19treatmentguidelines.nih.gov/whats-new/, accessed on 14 April 2021).

Identification and transmission tracking of potential antiviral resistant strains is essential for disease surveillance. Non-synonymous variants in *nsp12* have been reported in independent studies and different countries, particularly Nsp12:Pro323Leu [18,19], and in a single case associated with clinical failure of remdesivir treatment [20].

In this study, we provide a framework to monitor antiviral resistance emergence choosing RdRp-remdesivir as a proof of concept. We address the suitability of RdRp as a drug target for tackling SARS-CoV-2 by evaluating the selective pressure affecting RdRp and monitoring the emergence of potential escape mutations in key drug binding motifs. We screen real world data from both a global dataset consisting of more than 56,000 genomes from 105 countries, and an inpatient longitudinal genome cohort of 197 patients (189 untreated patients, eight remdesivir treated patients, of which three have been sequenced in follow-up time points). We show that RdRp is under negative selection and that potential escape mutations to remdesivir are rare in genomes available from globally circulating viral lineages. We further show that where potential escape mutations are present, they generally do not hamper RdRp stability, suggesting that compensatory mutations are not necessary.

## 2. Materials and Methods

### 2.1. Inferring Signatures of Selection in nsp12

We inferred selection on the *nsp12* gene using Tajima’s D test statistic, which is the comparison of average number of pairwise differences with the number of segregating sites, as well the ratio of nonsynonymous/synonymous (dN/dS) mutations per codon along *nsp12.* From the initial 77,150, 58,208 consensus sequences from GISAID passed the 10%Ns quality filter of COVGAPgenomes. These were further cleaned and filtered from redundant sequences (*n* = 614) using Sequence Dereplicator and Database Curator (SDDC) program [21]. Since selection analysis tools are limited in the number of sequences that can be handled, we down-sampled to represent all phylogenetic lineages from all countries and each month by 25 sequences using the nextstrain analysis pipeline v.2.0.0 (nextstrain.org) [22] resulting in 16,184 genomes that could be processed further. Whole-genome consensus sequences were aligned against NCBI Refseq sequence Wuhan-Hu-1 NC_045512.2 using MAFFT v7.467 (method FFT-NS-fragment; options--reorder--keeplength--mapout--kimura 1--addfragments--auto) [23]. The alignment was trimmed to the nucleotide region that codes for *nsp12* (PDB protein identifier 6M71) between 13442-16234, the final stop codon excluded as required for the analysis. Since *nsp12* is translated with a ribosomal slippage, in which the first nine amino acids are read in the second open reading frame (ORF) and the following amino acids in the first ORF, an extra cytosine (C) was included after position 27 to provide the information for all translated codons for subsequent analyses of selection. A last filtering step was applied before the final selection analysis to remove sequences with premature STOP codons (*n* = 12), remove sequences that contained gaps (-) or missing data (N) (*n* = 1141), as well as sequences with ambiguous characters (IUPAC codes: YKRWSMDVHB; *n* = 413). The selection analysis dataset finally contained 14,612 SARS-CoV-2 consensus sequences.

To infer the selection pressure that is acting on the entire *nsp12* gene region, we calculated Tajimas’s D statistic [24,25] using MEGA version 7 [26] to test for neutral selection for the entire coding sequence. The test statistic is based on two estimates, number of segregating sites [27] and average number of nucleotide differences, gained from pairwise comparisons. Tajimas’s D statistics equalling zero means that number of segregating sites roughly equals average nucleotide differences; Tajimas’s D being smaller than zero means that number of segregating sites are more abundant than average nucleotide differences, indicating an excess of rare alleles or a recent population expansion after a bottleneck; and Tajimas’s D being larger than zero means that fewer segregating sites than average nucleotide differences exist, indicating that rare alleles are selected against.

Further, we inferred the ratio of nonsynonymous to synonymous mutations (dN/dS or omega) in a Bayesian sliding window approach using genomegamap [28]. We ran 1,000,000 MCMC chains discarding the first 100,000 iterations (burn-in). The dN/dS ratio per codon is indicative of the kind of selection acting on the codons. Non-synonymous mutations are biologically selected against as they can result in structural changes and hence functional changes unless they provide selective advantage, meaning conveying larger reproductive success. An excess of non-synonymous mutations (dN/dS > 1) is interpreted as the site being under positive selection, whereas an excess of synonymous mutations (dN/dS > 1) is interpreted as purifying or balancing selection acting on this particular position. A balance of non-synonymous and synonymous mutations (dN/dS = 1) is understood as neutral selection. We set the priors of theta (the observed nucleotide diversity of the sample population) to 0.0006 and for kappa (per-path rate bias, from which the transition/transversion ratio can be calculated as kappa/2) to 2.3 as previously inferred in Wilson (2020) [28].

### 2.2. Global Dataset, Spatiotemporal Trends and Genome Entropy

77,150 SARS-CoV-2 consensus genomes were downloaded as of 12 July 2020 from the GISAID platform [29]. The genomes were quality filtered: 18,942 genomes were discarded as containing more than 10% ambiguous base calls (Ns); and 1402 genomes were discarded for containing ambiguous variant calls. Mutations were considered only when a clear alternative allele was found, and genomes reporting errors in the alternative allele calls were discarded. Degenerate base calls in the variant call other than N were not determining the exclusion of the genome from the dataset. Using our COVGAPgenomes pipeline [30], the sequences were first aligned to the Wuhan-1 reference [31] using mafft v7.467, point mutations were identified with snp-sites v2.5.1 [32]. The collected variants were then annotated using snpeff v4.5covid19 [33]. The variants positions, together with the variant annotation, were screened using R base. Genomes whose variants fell into the potential escape motifs were labelled as potential escape mutants.

Statistics on spatiotemporal trends were calculated via R base v3.6.2, lineages classification was inferred through Pangolin [34]. Data mining and structuring was performed through the R packages: tidyr v1.1.2, dplyr v1.0.2, reshape2 v1.4.4 [35], BiocGenerics v0.32.0 [36], IRanges v2.20.2 [37], Biostrings v2.54.0, XVector v0.26.0, S4Vectors v0.24.4, shapefiles v0.7, foreign v0.8-76. Plots were drafted using R packages: ggpubr v0.4.0, ggExtra v0.9, cowplot v1.1.0, lubridate v1.7.9 [38], rgeos v0.5-5, rnaturalearth v0.1.0, rnaturalearthdata v0.1.0, sf v0.9-5, sp v1.4-2, maps v3.3.0, ggspatial v1.1.4, ggplot2 v3.3.2 [39].

Calculation of genome diversity took place using the chao2 index [40] available in the R package fossil v0.4.0. The index value was calculated for each single genome, and further structuring in table was performed through the Matrix v1.2-18 R package.

### 2.3. Genome Drafting Tool and Availability

We generated COVGAPgenomes, an adaptation of our bioinformatic COVID-19 Genome Analysis Pipeline (COVGAP, see Online Methods; doi:10.1371/journal.ppat.1009374) to detect all non-synonymous SNPs affecting the defined potential escape motifs and potentially hampering the functionality of drugs targeting RdRp, and to screen sequence data for minor allele variants.

Of note, the COVGAPgenomes platform can be adapted to detect any relevant variant, including binding sites of new antiviral drugs. This allows us to rapidly screen genome collections and provide molecular epidemiological surveillance in real time or retrospectively.

### 2.4. Patients, Samples, and Diagnosis

#### 2.4.1. Basel University Hospital Single Time Point Open Cohort

All persons with a first-time positive SARS-CoV-2 PCR test between the 26 February (first case in Switzerland) and the 30 April 2020 were eligible for inclusion in the cohort. During this time period, 10,310 diagnostic tests were performed. Of the 690 patients meeting the eligibility criteria, 341 were female and 349 were male, with age ranging from 29 to 68 years (average 48.79 years).

#### 2.4.2. Longitudinal Cohort and Remdesivir Treatment

All hospitalized SARS-CoV-2 positive patients (diagnosed between 26 February and 30 April 2020), with a minimum of two or more positive tests were eligible for inclusion in the cohort. After exclusion of patients who did not give general informed consent, the cohort counted 197 patients (114 males, 83 females) with age ranging from 44 to 79 years (average 62.15 years). Of those patients, eight received 200mg of remdesivir on the first day, 100 mg/day the following days in intravenous infusion for 30–60 min. Three of the treated patients showed successful amplicon sequencing during or after the treatment.

#### 2.4.3. Diagnosis and Sequencing

The procedure followed to routine-diagnose patients and to sequence the Basel open cohort viral genomes is described in Stange et al., 2020 [30]. The region harbouring the two potential escape motifs was amplified from the samples from the longitudinal cohort using primers Fwd:AGGAATTACTTGTGTATGCTGCTGA and Rev:TAACATGTTGTGCCAACCACCA, resulting in a 701 bp fragment. The Illumina DNA Prep kit was used to generate sequencing libraries from the fragments libraries before sequencing on an Illumina NextSeq500. From all 197 patients, this resulted in 259 analyzable samples.

### 2.5. Statistics and Visualization

#### 2.5.1. Genome Diversity and Statistical Test

To calculate the diversity of each genome chao2 index was chosen because if its incidence-based nature [41]. Log values were used to ensure normal distribution. Given the large size disparity between the two groups (“None” and “Motif”), a Monte-Carlo approach was chosen. For both first and second motif (*n* = 115, *n* = 69 respectively), the corresponding no-escape mutant group (“None”) (*n* = 56,691, *n* = 56,737 respectively) was randomly subsampled without replacement in 10,000 groups each with *n* = 150. Each subgroup was tested against the same motif group through t-student test. Resulting p-values were adjusted following the Benjamini–Yekutieli correction [42], and subsequently merged using the sum of logs Fisher method [43]. The R package metap v = 1.4 was used.

#### 2.5.2. Associated Mutation Inference, Structural Fitting and Visualization

To infer potential escape-associated mutations, all the samples from the global cohort (*n* = 56,806) were labelled based on whether or not they showed at least one non-synonymous mutation in either potential escape motifs, respectively. From the initial 16,051 mutations detected over the entire cohort, only mutations appearing in both non potential escape and potential escape mutants were considered (*n* = 1056). Generalised linear models were used to evaluate the correlation between each point mutation and the potential escape/non-escape predictor. Lineage was added as additional predictive term only when the mutation appeared in multiple lineages. The response error was assumed to be distributed binomially. The retrieved *p*-value was then corrected for multiple testing using Benjamini-Yekutieli correction [42] and considered significant only for those mutations showing an adjusted *p* value < 0.05. In order to assess response to which the significant mutations were associated, we first calculated for each mutation the frequency in potential escape mutants divided by the frequency in non-escape mutants. We then defined mutations as potential escape-associated, only if the potential escape/non-escape frequency ratio was above the 95th percentile of the significant mutation ratio distribution. Conversely, mutations showing a ratio below the 5th percentile were considered non-escape associated. Percentiles were established according to Hyndman and Fan (1996) [44] in order to obtain median-unbiased quantiles. The glm function of R base stats package 3.6.2 was used. The retrieved associated mutations were fitted into the protein structure reported by Yin et al. [45] (PBD ID 7BV2). Visualisation and figure drafting took place with pymol v2.4.1 (The PyMOL Molecular Graphics System, Version 2.4.1 Schrödinger, LLC. New York, NY, USA) [46].

#### 2.5.3. Stability Analysis of Mutation Combinations

The effect of mutations on the stability of RdRp was calculated by using the FoldX 5.0 advanced protein design suite [47]. FoldX 5.0 has the capabilities to measure the stability changes of a protein structure model upon several mutations and is able to consider the interactions of the protein with RNA in its calculations, which is vital for an analysis in the RdRp system.

The input PDB files are identical to those downloaded from the PDB and were not otherwise pre-processed.

We used the FoldX command “BuildModel” and enabled the calculation of interactions between the protein and RNA. For every combination of protein structure and mutations, the calculations of Gibbs energies of protein folding were repeated five times, and the differences between respective wild types and mutated proteins were reported. We then calculated the average total Gibbs energy difference over the six protein structures of RdRp to have a final estimate of the stability difference between the wild type and the mutated protein.

To distinguish genuine stability predictions from noise, we filtered the results based on the reported standard deviations of FoldX energy calculations as described by Buss et al. [48]. The accuracy of FoldX is described to be dependent on the resolution of the investigated structures. Given that the resolution of the used protein structures ranges on the lower end with values between 2.5 and 2.9 Ångstrom, we used the conservative threshold of 1.78 kcal/mol.

Energy differences below this threshold were not considered to denote changes in the stability of the investigated protein.

To have a diverse perspective on the impact of the candidate resistance and compensatory mutations, a stability analysis was conducted on multiple experimentally resolved structures of the SARS-CoV-2 RdRp: 7CXM [49], 7AAP [50], 7BV2 [45], 7C2K [51], 6YYT [52], and 6M71 [53] (Supplementary Table S9). An identical approach was followed to investigate the stability changes in SARS-CoV-2 S protein, with the stability analysis being carried on the following experimental resolved structures: 7A94 [54], 7A95 [54], 7A96 [54], 7A97 [54], 7A98 [54], 7KJ4 [55], 7KJ3 [55], 7KJ2 [55], 6XRA [56] (Supplementary Table S9). The PDB files of these structures were directly downloaded from the Protein Data Bank [57].

## 3. Results

### 3.1. Selection Analysis of nsp12

Understanding selection pressure on viral genes is critical for studying the potential effects of antiviral drugs. Therefore, we inferred the selection pressures on the whole *nsp12* gene (2793 nucleotides, 931 codons) applying Tajima’s D [24] and on the individual codons within the coding sequence assessing dN/dS. In a globally subsampled (14,612 sequences) dataset of SARS-CoV-2 *nsp12*, we identified 678 segregating nucleotide sites (24.3% variable sites). Most codons are conserved except for the amino acid position 323 (nucleotide position 967–969), where proline is often substituted for leucine. We then estimated the degree of genetic variation among the population, and we found a nucleotide diversity (pi) of 0.000389, a population nucleotide diversity (theta) of 0.0238, and a transition/transversion ratio of 1.94. Tajima’s *D* test statistic for *nsp12* was negative (D = −2.62), indicating an excess of rare alleles in this dataset. The dN/dS analysis of *nsp12* gives a mean dN/dS of 0.61. In more detail, along the gene, few codons (357) are within the credibility range for neutral selection, and most codons (574) have a dN/dS smaller than 1, indicating different degrees of purifying selection acting on the individual codons along the gene (Supplementary Figure S3). These results indicate that *nsp12* is under purifying selection, implying that accumulation and fixation of mutations is evolutionarily unfavoured with deleterious mutations being eliminated from the coding sequence.

### 3.2. Identification of Key Residues and Motifs for Drug Binding Sites

The RdRp complex involves the subunit Nsp7 and Nsp8 as stabilizer of the catalytic subunit Nsp12. The active site of Nsp12 consists of seven motifs (A to G). Motif C and residues Asp760, Asp761 constitute the catalytic active center, while motif F and G are both involved in RNA template and primer binding with residues Lys545 and Arg555 contacting the incoming bases [45]. Val557 is directly interacting with the RNA, while Phe480 is buried in the hydrophobic core (Supplementary Figure S1A,B). We focussed on the motifs encompassing the latter two residues along the following strategy: for Val557 we included all the neighbouring secondary structures at the interface with the catalytic site: the entire beta-sheet Val557 finds itself in, and two loose loops and part of the neighbouring alpha helix closer to the active site [53] (Supplementary Figures S1A,C and S2A). For Phe480, as seemingly no neighbouring structure binds either the template or the nucleoside, we included the entire loose loop and alpha helix neighbouring Phe480, as part of the hydrophobic core of RdRp and crucial for the protein proof-reading stability [4] (Supplementary Figures S1B,D and S2A). Of note, the single mutation associated with failure in remdesivir treatment is included in the selected motif [20]. The resulting motifs stretch from Nsp12:Arg467 till Nsp12:Val493 for the first motif (neighbouring Phe480), and Nsp12:Leu544 to Nsp12:Gln570 for the second motif (neighbouring Val557), referred to as first and second potential escape motifs respectively, for ease of readability (Supplementary Figure S2A). Mutations in the first potential escape motif are expected to lead to a proofreading change in the protein, while mutations in the second are expected to impact the binding affinity with RNA.

### 3.3. Potential Escape Mutants in the Global Dataset

Building on our published genome analysis pipeline, we developed an updated version, which is able to track nucleotide and ammino acid diversification of drug target residues, including the potential escape motifs in RdRp subunit Nsp12.

**Figure 1:**
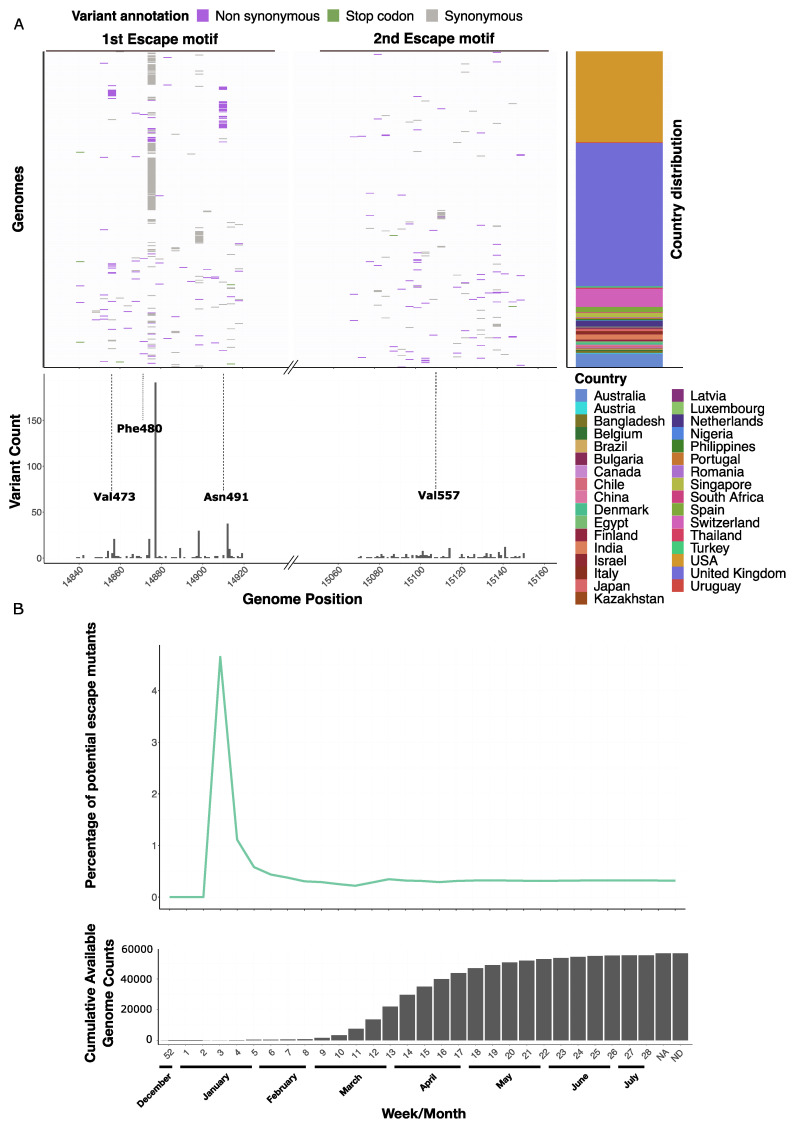
Potential escape mutations inside 1st and 2nd escape motif are not uniformly distributed, they are rare worldwide and stable overtime: (**A**) Mutation prevalence in the two potential escape motifs, SNPs are highlighted in the upper genome panel, their frequency in the lower variant count panel, the genome origin is depicted in the upper right country distribution panel. (**B**) Frequency changes of potentially resistant genomes over time become stable after the 13th calendar week in 2020 and settle to 0.32%. The cumulative count panel displays available genomes on GISAID at a defined week. The time frame considered spans from 25 December 2019 till 12 July 2020, NA indicates no date information available, ND indicates incomplete date information available.

56,806 high-quality publicly available SARS-CoV-2 genomes were collected between 24 December 2019 and 12 July 2020 from GISAID [29] (Supplementary Table S1). Non-synonymous mutations in the *nsp12* coding sequence were found in 46,469 of the 56,806 (81.08%) viral genomes. However, most of these mutations (e.g., position C14408T leading to Pro323Leu; Supplementary Figure S4) were neither part of the potential escape motifs nor located around key residues of the active site (Supplementary Figure S1). Only 182/56,806 (0.32%) genomes contained non-synonymous mutations within the potential escape motifs, which provided a total of 85 different mutations as potential candidates for reduced remdesivir effectiveness (Supplementary Tables S2 and S3) (Figure 1A).

Of the 182 genomes, three exhibited non-synonymous mutations affecting Nsp12:Phe480 (Nsp12:Phe480Leu, Nsp12:Phe480Ser and Nsp12:Phe480Cys). The residue Nsp12:Val557 was found to be mutated in a single genome (Nsp12:Val557Glu). Additional high frequency non-synonymous SNPs included those encoding Nsp12:Asn491Ser (occurring in 34 genomes) and Nsp12:Val473Phe (19 genomes). (Figure 1A, Supplementary Table S3).

The first genome carrying a non-synonymous variant falling into a potential escape motif (Nsp12:Ser564Ile) was registered on 20 January 2020 in a 30 year old female patient from China [58]. The sample carrying Nsp12:Phe480Leu was collected on 3 March 2020 from a 72 year old male patient from England (Figure 1A). With increasing numbers of available sequences, genomes carrying non-synonymous mutations in potential escape motifs settled at 0.21% by calendar week 13 and reached a stable rate of 0.3% (±0.064) from week 15 on (available genomes *n* = 35,055). No proportional increase has been detected in any time points after that date, even after conditional approval of remdesivir (Figure 1B). To investigate the geographical distribution of the potential escape mutants, we considered only the countries having submitted at least 100 high quality genomes. We found that Switzerland (2.2%), Chile (1.4%), and Bangladesh (1.02%) showed the highest percentages of genomes that feature potential escape mutations (Supplementary Figure S2B and Table S4).

To add granularity to the genome data derived from Switzerland, we collected an open cohort of 690 individuals from the University Hospital Basel, who were tested only once (single-time tested) between 23 February and 30 April 2020. We did not find any mutation in the potential escape motifs, nor minority alleles in the samples that would hint at intra-host diversity. The variant distribution across RdRp is in line with the distribution observed in the global dataset, with the exception of a high frequency of a synonymous mutation in nucleotide position 15,324 (located in RdRp), recently described within a Basel-area specific lineage [30] (Supplementary Figure S5).

We determined phylogenetic (pangolin nomenclature) lineages of the genomes carrying potential escape motif mutations [34], and found that they are distributed across 37 different lineages in 21 countries. Among these is the B1.108 lineage (32 genomes), only identified in the USA, first detected on 14 March 2020 and not observed after 24 April 2020. This lineage is defined by nucleotide mutation *nsp12*:A14912G, encoding a non-synonymous SNP leading to Nsp12:Asn491Ser. This lineage thus shows potential escape mutations in all its isolates (Supplementary Table S5). These results indicate that potential escape mutations, although potentially they might be promptly selected for, rarely emerge into commonly circulating lineages, independently of geographical location.

### 3.4. Potential Escape Mutants during Remdesivir Treatment

**Figure 2:**
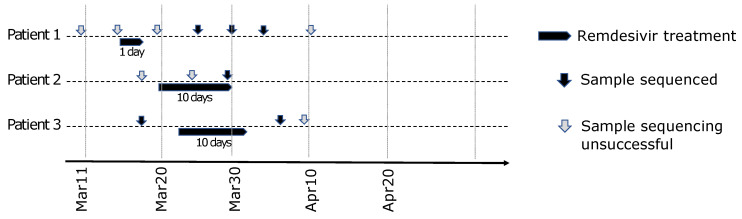
Chronology of remdesivir treatment in three local patients in the study period. Successfully sequenced samples are marked with black arrows. Grey arrows indicate samples that could not be sequenced for reasons of accessibility, sample quality or low viral load. Remdesivir treatment is indicated by black horizontal bars.

From mid-March 2020, University Hospital Basel participated in COVID-19 clinical trial NCT04323761 on remdesivir treatment for COVID-19 pneumonia. We collected samples from a longitudinal cohort of 197 SARS-COV-2 positive hospitalized patients from 26 February until 30 April 2020. In order to gain sequencing coverage on the cohort, all the time points from the 197 patients (259 samples in total) were amplicon sequenced focussing on the potential escape motifs in RdRp (bases 14,545 to 15,246). A total of eight patients were treated with remdesivir (duration between 1 and 10 days, first day bolus of 200 mg followed by 100 mg daily), and for three of those sequencing was successful during or after the treatment (Figure 2) (Supplementary Table S6). Through our genome analysis pipeline (COVGAP), we automatically monitored the occurrence of major (high alternative frequency) and minor (low alternative frequency) SNPs causing amino acid changes in any specific motifs.

We did not observe any highly supported variants in the two potential escape motifs in the remdesivir-treated patients. However, in one sample from a hospitalised 47 year-old male patient, who did not receive remdesivir treatment, we observed a minority allele carrying a non-synonymous mutation in *nsp12* encoding Thr374Cys (21% alternative allele support of 27 read coverage). This substitution is not within the potential escape motifs and is outside the active site of RdRp. These findings are in line with the rarity of potential escape mutants observed in the global dataset, which support the hypothesis of a low selective pressure provided by remdesivir treatment.

### 3.5. Associated Mutations and Stability Loss

**Figure 3:**
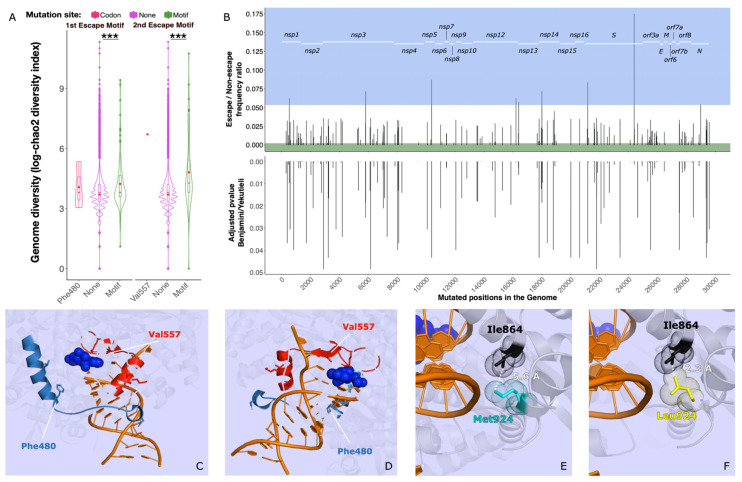
**Mutations in escape motifs harbour higher genome nucleotide and tightly associated mutations:** The genome entropy is significantly higher in 1st and 2nd motif escape mutants. (**A**) Diversity is calculated as incidence-based mutation richness along the chao2 diversity index, boxplot represents the interquartile range, red dots indicate the mean. Stars indicate the merged p-value with *** indicating a *p*-value < 0.001. Significance was calculated with a Monte Carlo t-test simulation, see methods. (**B**) Association between escape mutants and other mutations across the genome. Candidates are evaluated through generalised linear models fit with lineage correction. Upper panel: depiction of mutation incidence ratio escape/non escape. Only mutations showing a ratio > 95th percentile are considered escape-associated (light blue area), of note mutation in position 16,210 is significantly associated to escape mutants. Lower Panel: adjusted p-value for multiple testing according to Benjamini-Yekutieli—only mutations with significant pvalues are shown. (**C**,**D**) Location of escape mutations on RdRp bound to RNA template (in orange) and remdesivir (in electric blue), 1st escape motif is indicated in blue, 2nd escape motif is indicated in red. (**E**,**F**) Met924Leu (encoded by a SNP in position 16,210) decreases the distance to Ile864 by more than 2-fold. Panel (**E**) depicts the original residue Met924 (in cyan) while panel (**F**) depicts the in silico mutated residue Leu924 (in yellow) Cartoons and atomic structures are generated through the Pymol software on the protein crystal structure PDB ID: 7BV2 [45].

Escape mutations often result in a remarkable destabilization of the protein, leading to fitness loss [59]. Compensatory mutations are those mutations co-occurring (associated) with escape mutations, rescuing that stability loss [59]. We addressed the presence of a compensatory trade-off by analysing first the nucleotide diversity. If such an association was present, a higher number of mutations should be present in genomes carrying potential escape mutations. We compared the nucleotide diversity of entire genomes containing vs. non containing potential escape mutations (potential escape mutants vs. non-escape mutants respectively) (Figure 3A). We found that the level of nucleotide diversity was significantly higher for potential escape mutants compared to non-escape mutants for both potential escape mutants genomes type after t-test run over Monte-Carlo simulation (chi-sq = 115,989.4, df = 20,000, merged-pval << 0.001) and (chi-sq = 191,521.7, df = 20,000, merged-pval << 0.001), (Figure 3A) (Chao2 diversity incidence index; Supplementary Table S1). This result points towards the presence of mutations co-occurring (associated) with potential escape mutations.

We then screened the potential escape mutant genomes to identify those mutations associated with potential escape mutations. Within the 182 genomes featuring potential escape mutations, we discarded five as they introduced stop codons (i.e., unviable) and we identified over the entire genome a total 1056 non-synonymous mutations, including the 85 potential escape mutations. We evaluated the actual co-occurrence of the 971 non-escape mutations with the 85 escape mutations through a generalized linear model. After correcting for lineage grouping, 174 non-synonymous mutations showed significant response to the predictor (Figure 3B, Supplementary Table S7). Among these, nine were positively associated with potential escape mutations.

Only one of the potential escape-associated mutations found falls into *nsp12*, at genome position 16,210 (Nsp12:Met924Leu) (Supplementary Table S8). This variant occurs in multiple genomes from various lineages carrying the potential escape mutations Nsp12:Arg555Pro, Nsp12:Met566Val, Nsp12:Thr567Ser, and Nsp12:Arg569Gly. Structural modelling suggests that the substitution to Leu924 shortens the distance with the close Ile864 residue from 5.6 Å to 2.3 Å and could therefore hamper the binding to the RNA template [45] (Figure 3E,F).

To ultimately address whether the potential escape mutations and their associated mutations have a compensatory nature, we determined the protein stability change for RdRp carrying various combinations of potential escape and escape-associated mutation combinations. For each scenario we calculated the energy state and evaluated whether potential escape associated mutations rescue a stability loss triggered by potential escape mutations (Supplementary Table S9). We included the combinations between potential escape mutations Nsp12:Arg555Pro, Nsp12:Met566Val, Nsp12:Thr567Ser, Nsp12:Arg569Gly, Nsp12:Val473Phe, and the associated mutation Nsp12:Met924Leu (Non mutated residues in Supplementary Figure S1). As a control we evaluated Nsp12:Phe480Leu and Nsp12:Val557Leu (Supplementary Table S9). To correct for possible poor protein resolution, we inferred each combination on six different potential RdRp structures (Supplementary Figure S6 and Table S9).

We detected significant destabilisation only in the case of Nsp12:Met566Val (2.53 kcal/mol) and Nsp12:Val473Phe (3.97 kcal/mol). The former is associated with Nsp12:Met924Leu, which does not show any sort of stabilising effect on its own, nor in combination with any other potential escape mutation. Nsp12:Val473Phe is also strongly associated with a SNP located at genomic position 24,378 (S:Ser939Phe, adjusted *p* value = 0.00012, Supplementary Table S7).

The spike protein S, which mediates host receptor binding, is the main target of the immune response. The presence of S:Ser939Phe may cause an impact on the host cell binding and entry.

Interestingly, while Ser939Phe yields no effect on pre-fusion S structures (−0.17 kcal/mol), this substitution yields a stabilising effect on post-fusion S structures (−1.9 kcal/mol) (Supplementary Table S9). The physiological gain of this stabilisation is yet to be understood. These results show that potential escape-associated mutations within RdRp are unlikely to compensate for RdRp stability losses.

## 4. Discussion

The rapid global spread of SARS-CoV-2 has led to the emergence of many different variants in *nsp12*. We designed our workflow aiming to potentially screen any hotspot of resistance emergence. As a proof of concept, we offer an evolutionary perspective on RdRp-remdesivir interaction. Our analysis of SARS-CoV-2 RdRp evolution on a global scale demonstrates the negative selection pressure acting on the protein. RdRp mutations potentially linked to remdesivir resistance are rare in the global dataset, and we did not observe consistent impact of these mutations on protein stability. Seemingly, none of the mutations identified has yet provided a selective advantage for circulating viral lineages under current pressures. In particular, an Nsp12:Pro323Leu mutation was identified in SARS-CoV-2 genomes from infected patients collected from December 2019 to mid-March 2020 but had no reported consequence for RdRp active site [18]. This observation is in line with the present finding of negative selection pressure on *nsp12*, thereby not favouring resistant variants of SARS-CoV-2. Since mutations in the potential escape motifs of RdRp have been shown to exert a fitness cost for correspondent related viruses [13], such variants in SARS-CoV-2 would not be expected to propagate efficiently, unless the virus were under constant challenge by antiviral drug treatment targeting RdRp.

Remdesivir efficacy has been demonstrated previously in SARS-CoV throughout in vitro studies of infected cell cultures [60,61,62], as well as in rhesus macaque models [63]. However, its clinical usage against SARS-CoV-2 has yielded contradictory evidence [14,16]. In the global dataset, the first samples carrying remdesivir potential escape mutations were collected on the 20 January in China [58], and 3 March in the United Kingdom, far before the introduction of remdesivir as a provisionally authorised medicament, (first approval: 1 May 2020 [64]). Those two early mutations did not further spread, as there is no trace in later time points in the global dataset, likely owing to purifying selection.

Remdesivir treatment for COVID-19 was initiated at our hospital as a part of a clinical trial in mid-March 2020; it was approved for general use in Swiss hospitals as of 30 June 2020. Among the patients enrolled in the trial, only eight fulfilled the criteria for treatment with remdesivir within the study period, and for three patients only we obtained SARS-CoV-2 escape motifs amplicons during or after treatment. The only mutation affecting RdRp identified was located just outside one of the potential escape motif, and was found in a non-remdesivir treated patient. Although the low amount of evaluated patients could be the reason of these results, another potential explanation could be that remdesivir treatment, at the administered doses, does not provide enough selective pressure against the virus due to a low efficacy [65].

We observed that potential escape mutations do not seem to be correlated to RdRp destabilization, and if this is true for some exceptions, escape associated mutations do not seem to rescue such loss. If the potential escape mutations recovered were costly in terms of fitness, we would have expected to observe a destabilizing effect on RdRp, and a corresponding stabilizing effect from potential escape-associated mutations. This is surprisingly not the case, especially considering Nsp12:Arg555Pro, a key residue involved in remdesivir binding, and Nsp12:Val557Leu [45,53].

Among the potential escape mutations showing stable association with other variants, the only examples leading to destabilization of RdRp are Nsp12:Met566Val and Nsp12:Phe480Leu. We found that Nsp12:Met924Leu, the only significantly associated mutation on RdRp, does not rescue Nsp12:Met566Val destabilization, nor does it have a stabilizing effect on its own.

These results, however, do not exclude that a different form of fitness loss/compensation not involving stability could take place [66]. For example, Nsp12:Arg555Pro could disrupt the binding affinity of remdesivir and Nsp12:Met924Leu could improve the protein binding to the template. In other motifs of RdRp, a depolarizing mutation such as Nsp12:Met924Leu has been linked to a reduction of the polymerase proofreading activity [4]. Alternatively, such an association could involve the viral physiology more widely, as for example the association between the potential escape mutation Nsp12:Val473Phe and S:Ser939Phe. The nature of such cross-protein association remains speculative and additional data would be necessary to illustrate that in full.

The rarity of potential escape mutations in globally circulating SARS-CoV-2 lineages coupled with the purifying selection acting on RdRp could indicate that remdesivir use has not yet selected for resistant variants observable on a global scale. However, any selective pressure caused by remdesivir will not necessarily be captured within the entire global dataset (*n* = 56,806), as available sequences represent (I) largely samples from non-remdesivir treated patients (data available from the GISAID repository) and (II) likely the first isolate from patients prior to treatment with remdesivir. As such, it will be important to continuously sequence isolates from antiviral treated patients and monitor for the evolution and transmission of potential escape mutations.

## 5. Conclusions

In summary, our study offers a framework for the surveillance of SARS-CoV-2 evolution focusing on the potential emergence of antiviral resistance. Our findings demonstrate the purifying selection of RdRp worldwide and point towards a low selective pressure provided by anti-RdRp drugs (e.g., remdesivir). Potential remdesivir escape mutations were very rare in the global genomic dataset, which could be an indicator of little selective pressure, and hence no impact on the therapeutic efficacy of remdesivir. Notably, our analysis could be extended to other repurposed RdRp-targeting drugs, such as sofosbuvir and ribavirin, that have the same mechanism of RdRp inhibition as remdesivir. While our study provides a starting framework to draw hypotheses about resistance emergence, further proteomics experiments are needed to corroborate our finding on the identification of escape-associated mutations potentially destabilizing RdRp. Finally, our data supports SARS-CoV-2 RdRp as a viable drug target candidate, although we suggest that patients treated with antiviral drugs should be closely monitored for the potential emergence of antiviral resistance.

## 6. Ethics

The study was conducted according to good laboratory practice and in accordance with the Declaration of Helsinki and national and institutional standards and was approved by the ethical committee (Ethikkommission Nordwest und Zentralschweiz, www.eknz.ch, accessed on 9 April 2020; ID number 2020-00769, approved the 9 April 2020). The clinical trial accession number is NCT04351503 (clinicaltrials.gov). 

## Data Availability

Genome assemblies from the Basel open cohort were submitted to GISAID. Amplicon sequences from the remdesivir treated patients are available upon request. The code inherent to COVGAP and COVGAPgenomes can be found at https://github.com/appliedmicrobiologyresearch, accessed on 18 May 2021. The scripts and code used to draft the analyses of this study can be found at https://github.com/appliedmicrobiologyresearch/SARS-CoV-2_RdRp accessed on 18 May 2021.

## Supplementary Materials

The following are available online at https://www.mdpi.com/article/10.3390/microorganisms9051094/s1, Figure S1: Key residues in RdRp structural context. A–B (180° rotation): the vast majority of key amino acids (Val473, Phe480, Asn491, Arg555, Val557, Met566, Thr567 and Arg569) lies on the fingers region, with the exception of Met924 (thumb), and Pro323 (interface). C–D (180° rotation): Close-up of remdesivir binding site with highlight of the conserved RdRp motifs (A to G). Notably, the key residue Arg555 is located on the conserved motif F. Cartoon, surface and atomic structures were generated through PyMol on the crystal structure from PDB: 7BV2 [45] Figure S2. A: Remdesivir interaction with RdRp active site, potential escape motifs, Graphic interface was achieved through Biorender.com B: Motif distribution over country. Cartoon and atomic structures were generated through PyMol on the crystal structure from PDB: 7BV2 [45] Figure S3: Codon-based estimates of the ratio of nonsynonymous to synonymous mutations (omega, dN/dS) along *nsp12* using a sliding window model in a Bayesian computation approach, Figure S4: Mutation distribution in RdRp shows hotspots of variants mostly not affecting the binding site of template and remdesivir, Figure S5: Basel open cohort: mutation distribution in RdRp is in line with the global trend, except for a mutation with high prevalence in the larger Basel area at genome position 15324. Figure S6: Average energy difference between wild-type and mutated RdRp across six different experimental structures, the noise detection threshold of 1.78 kcal/mol was established explained in the methods section Table S1: Global data set metadata per sample as of 12.07.2020, Table S2: Global samples presenting escape mutations in one of the two escape motifs as of 12.07.2020, Table S3: Point mutations (SNPs) occurring in escape mutants, Table S4: Number of escape mutants per country, only countries submitting more than 100 genomes were considered. Table S5: Occurrence of escape mutations per lineage, positive and negative designate the presence of mutations in one of the two escape motifs, or in none of them, respectively, Table S6: Occurrence and duration of remdesivir treatment in hospitalized patients. Table S7: Generalized linear models define the association between escape mutations and other mutations co-occurring in the genome. Table S8: Global samples containing predicted escape mutations and escape-associated mutations. Table S9: Stability measures under differential co-occurrence of escape mutations and escape associated mutations.
