# Supplementary material for: Global Genomic Analysis of SARS-CoV-2 RNA Dependent RNA Polymerase Evolution and Antiviral Drug Resistance
Source: Microorganisms. 2021 May 19;9(5):1094. doi: 10.3390/microorganisms9051094 (PMC8160703; doi:10.3390/microorganisms9051094)
Supplement: Supplementary file 1 [file microorganisms-09-01094-s001.zip › Supplementary_files1/SI_Figure2.pdf]

A

B

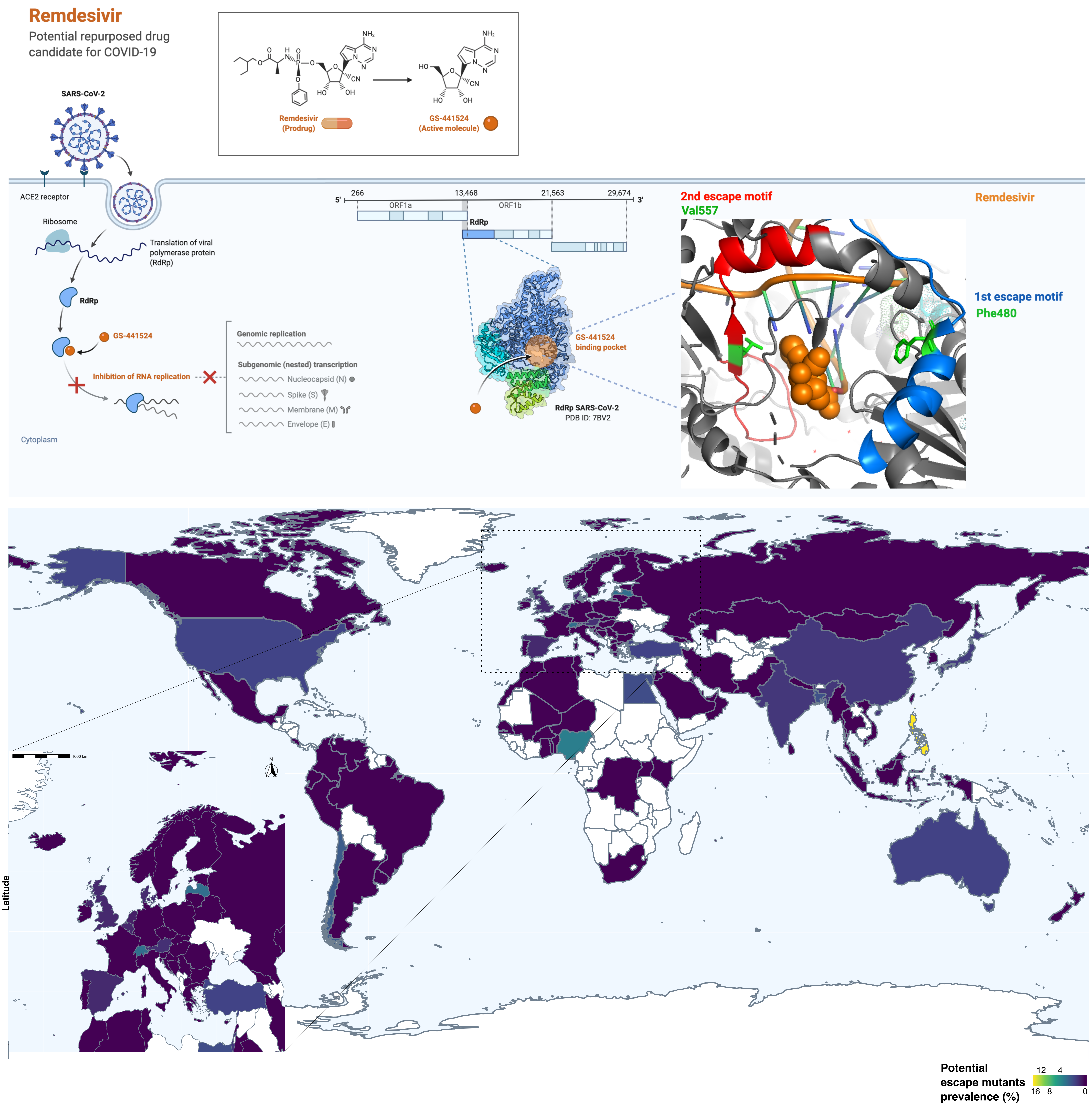
Remdesivir
Potential repurposed drug candidate for COVID-19
Remdesivir (Prodrug)
GS-441524 (Active molecule)
SARS-CoV-2
ACE2 receptor
Ribosome
Translation of viral polymerase protein (RdRp)
RdRp
GS-441524
Inhibition of RNA replication
Genomic replication
Subgenomic (nested) transcription
Nucleocapsid (N)
Spike (S)
Membrane (M)
Envelope (E)
Cytoplasm
5'
266
13,468
21,563
29,674
3'
ORF1a
ORF1b
RdRp
GS-441524 binding pocket
RdRp SARS-CoV-2
PDB ID: 7BV2
2nd escape motif
Val557
Remdesivir
1st escape motif
Phe480
1000 km
N
Latitude
Potential escape mutants prevalence (%)
12
4
16
8
0
