# Supplementary material for: Global Genomic Analysis of SARS-CoV-2 RNA Dependent RNA Polymerase Evolution and Antiviral Drug Resistance
Source: Microorganisms. 2021 May 19;9(5):1094. doi: 10.3390/microorganisms9051094 (PMC8160703; doi:10.3390/microorganisms9051094)
Supplement: Supplementary file 1 [file microorganisms-09-01094-s001.zip › Supplementary_files1/SI_Figure5.pdf]

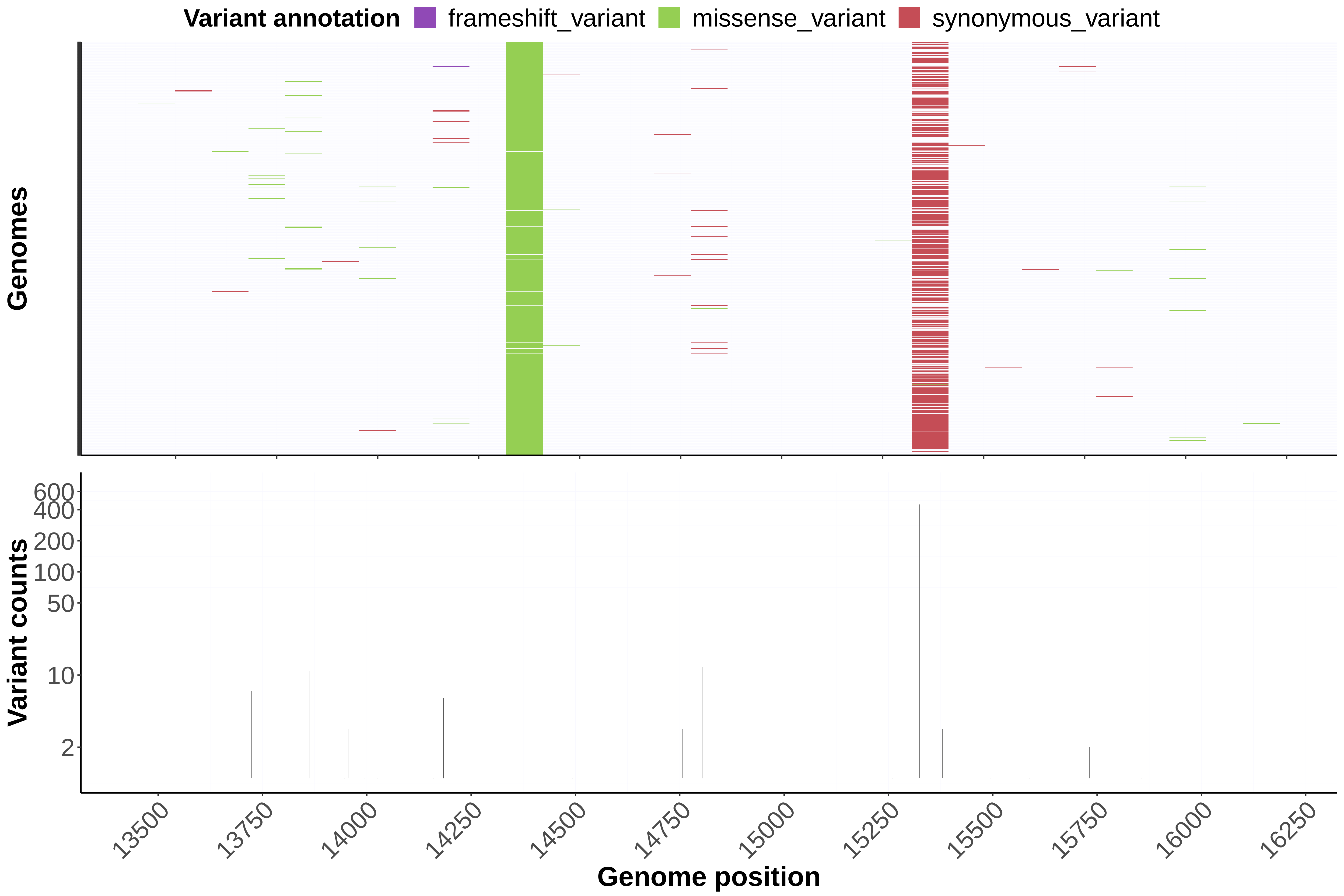
Variant annotation
frameshift_variant
missense_variant
synonymous_variant
Genomes
Variant counts
600
400
200
100
50
10
2
13500
13750
14000
14250
14500
14750
15000
15250
15500
15750
16000
16250
Genome position
