# Supplementary figures and images for: Global Genomic Analysis of SARS-CoV-2 RNA Dependent RNA Polymerase Evolution and Antiviral Drug Resistance
Source: Microorganisms. 2021 May 19;9(5):1094. doi: 10.3390/microorganisms9051094 (PMC8160703; doi:10.3390/microorganisms9051094)

A

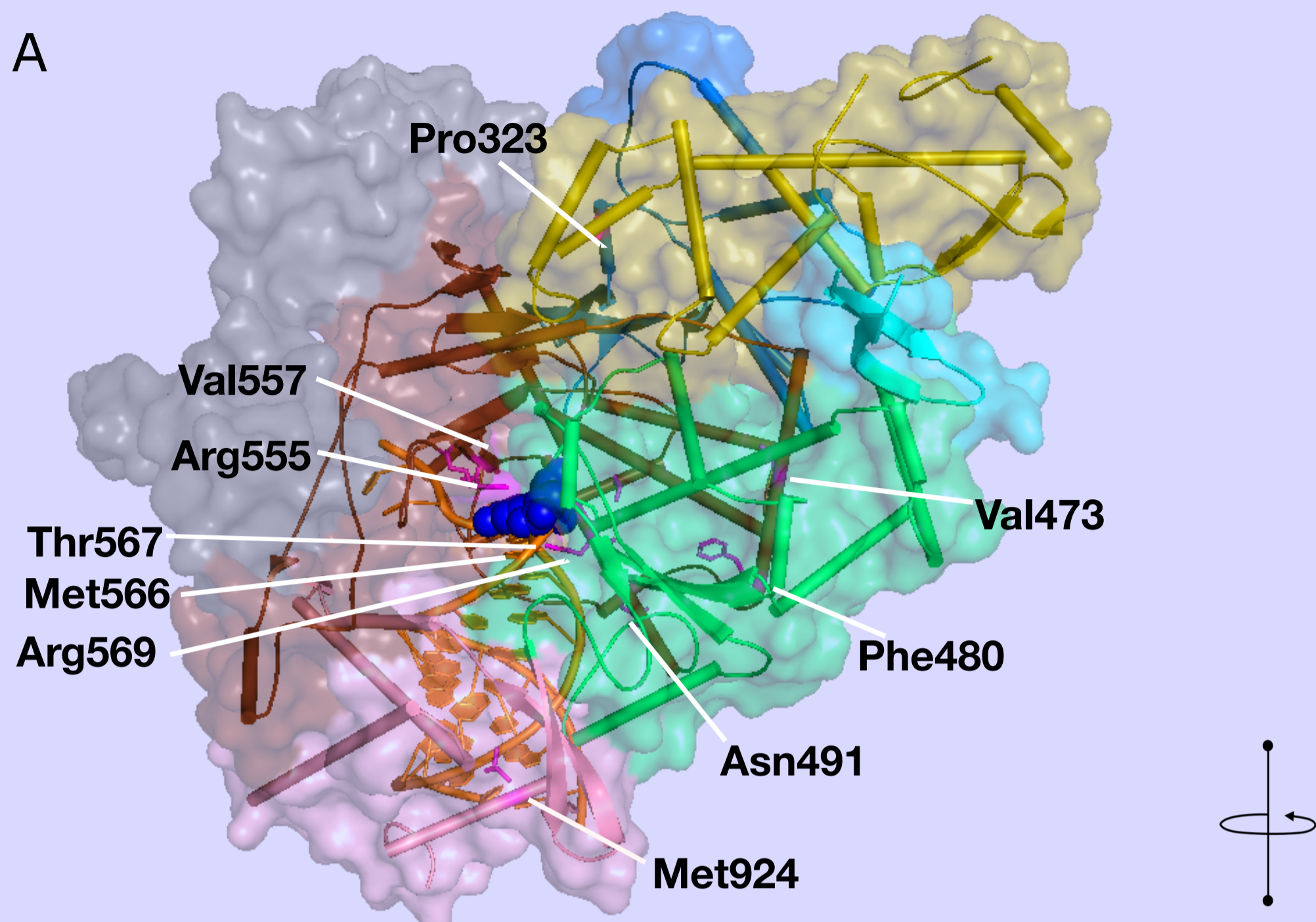

B

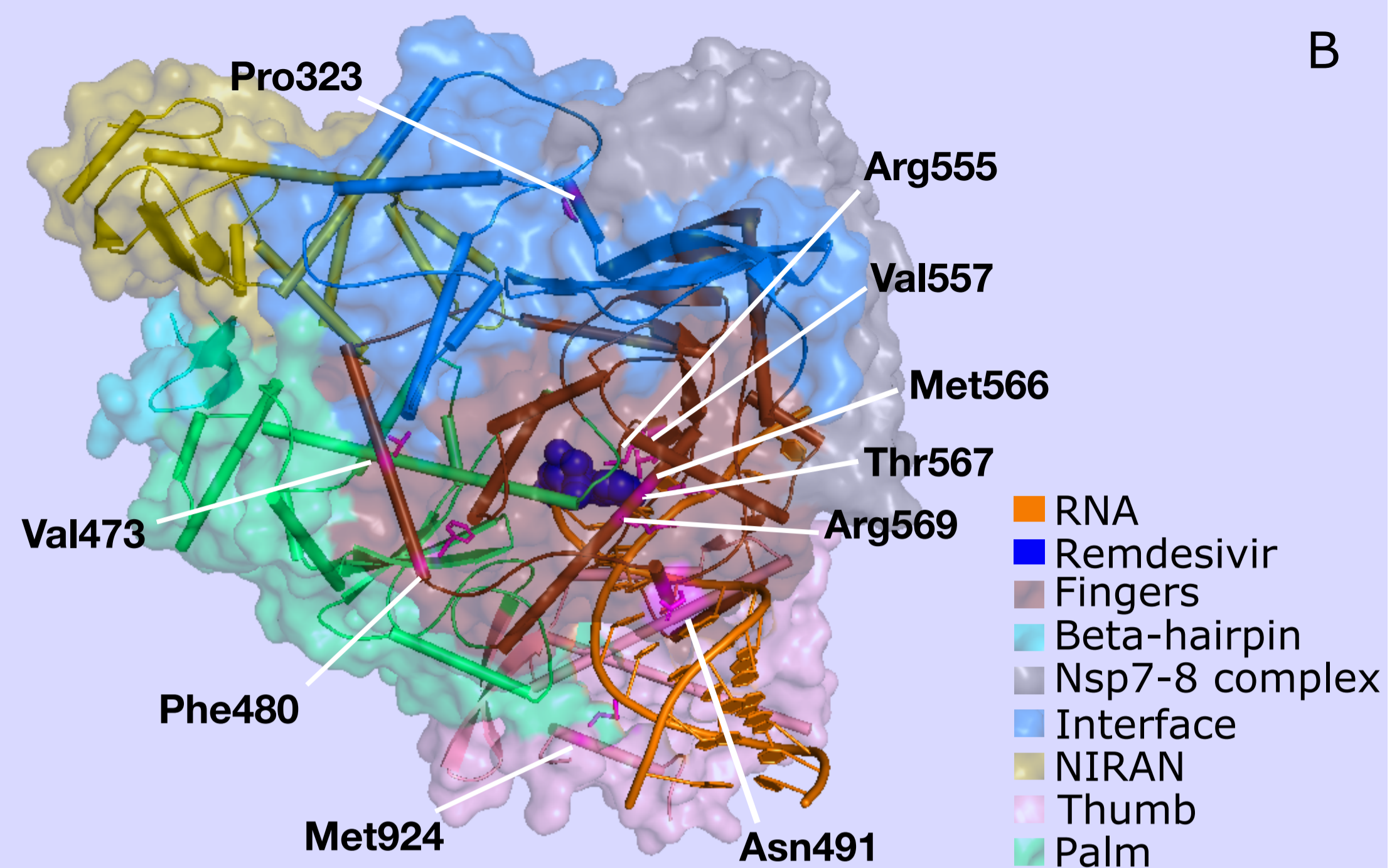

C

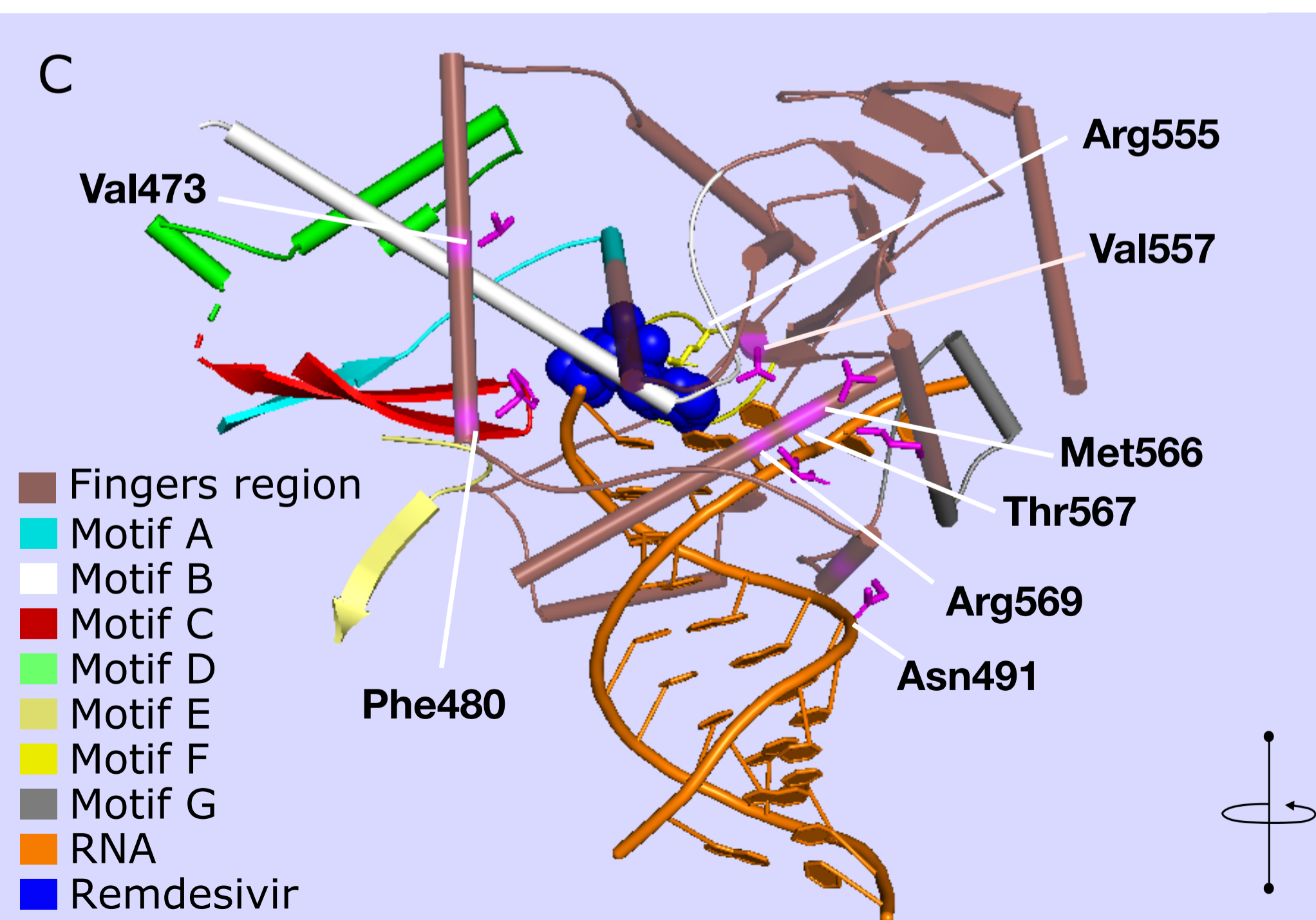

D

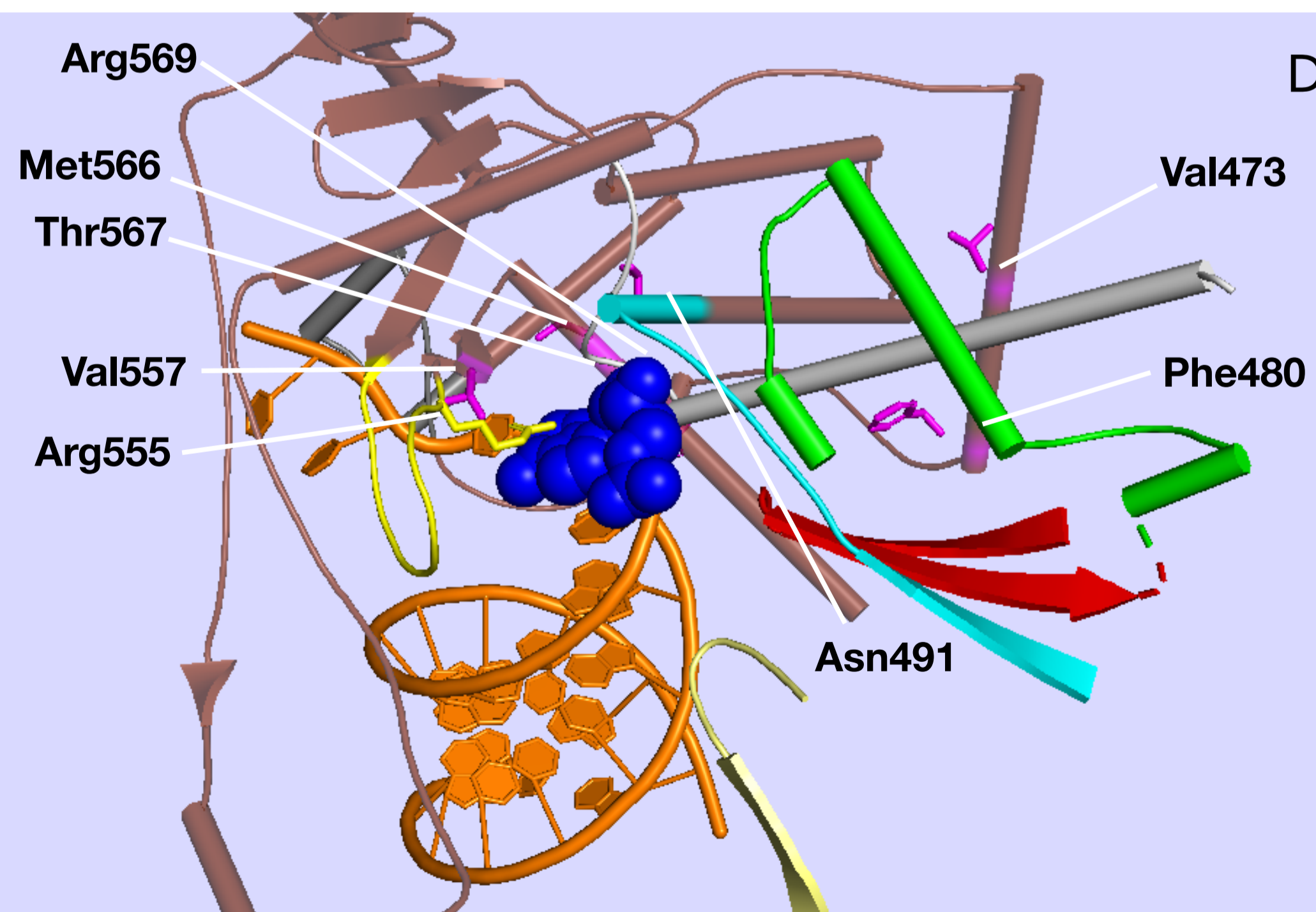

Supplement: Supplementary file 1 [file microorganisms-09-01094-s001.zip › Supplementary_files1/SI_Figure1.pdf]

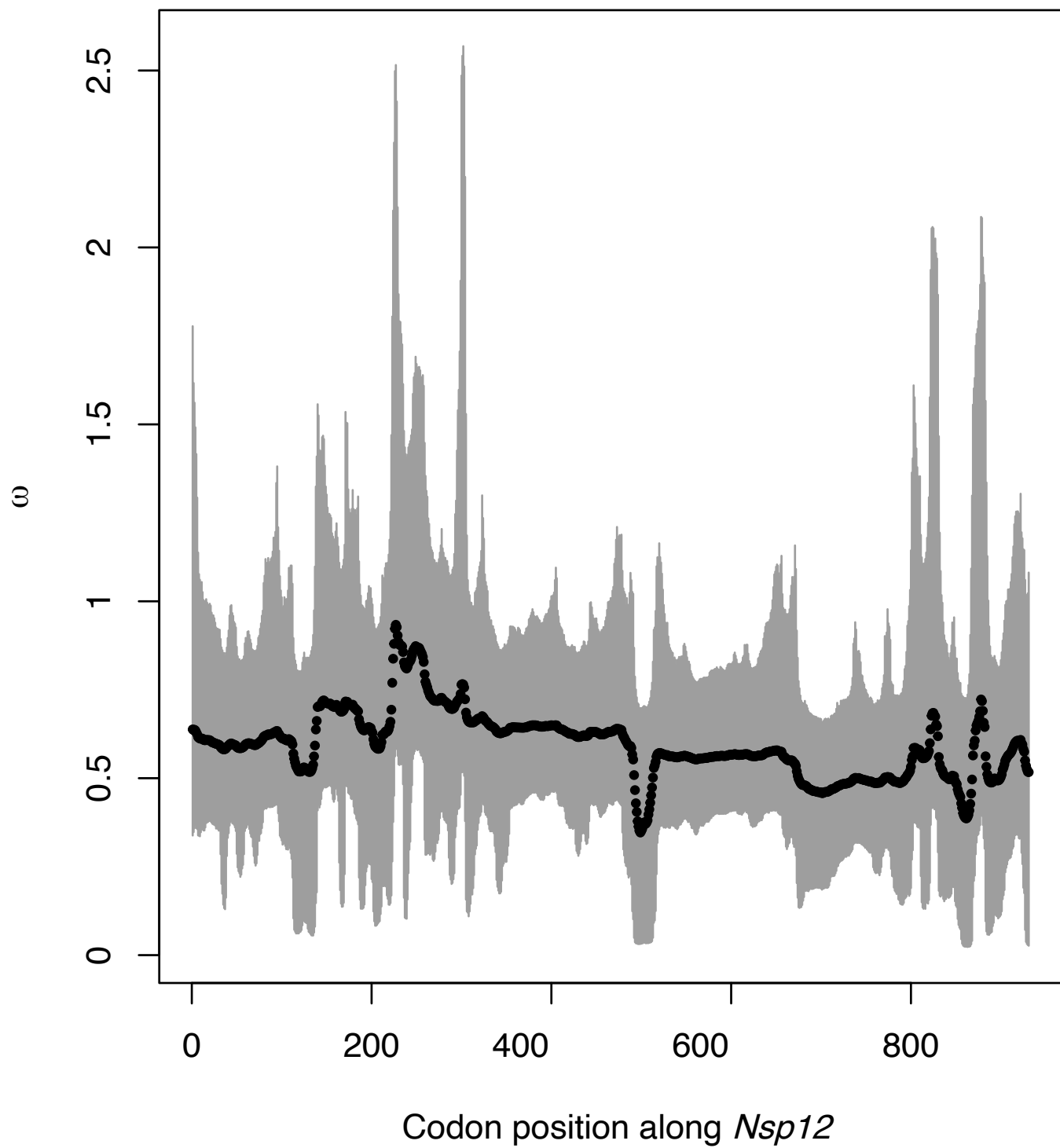

Supplement: Supplementary file 1 [file microorganisms-09-01094-s001.zip › Supplementary_files1/SI_Figure3.pdf]

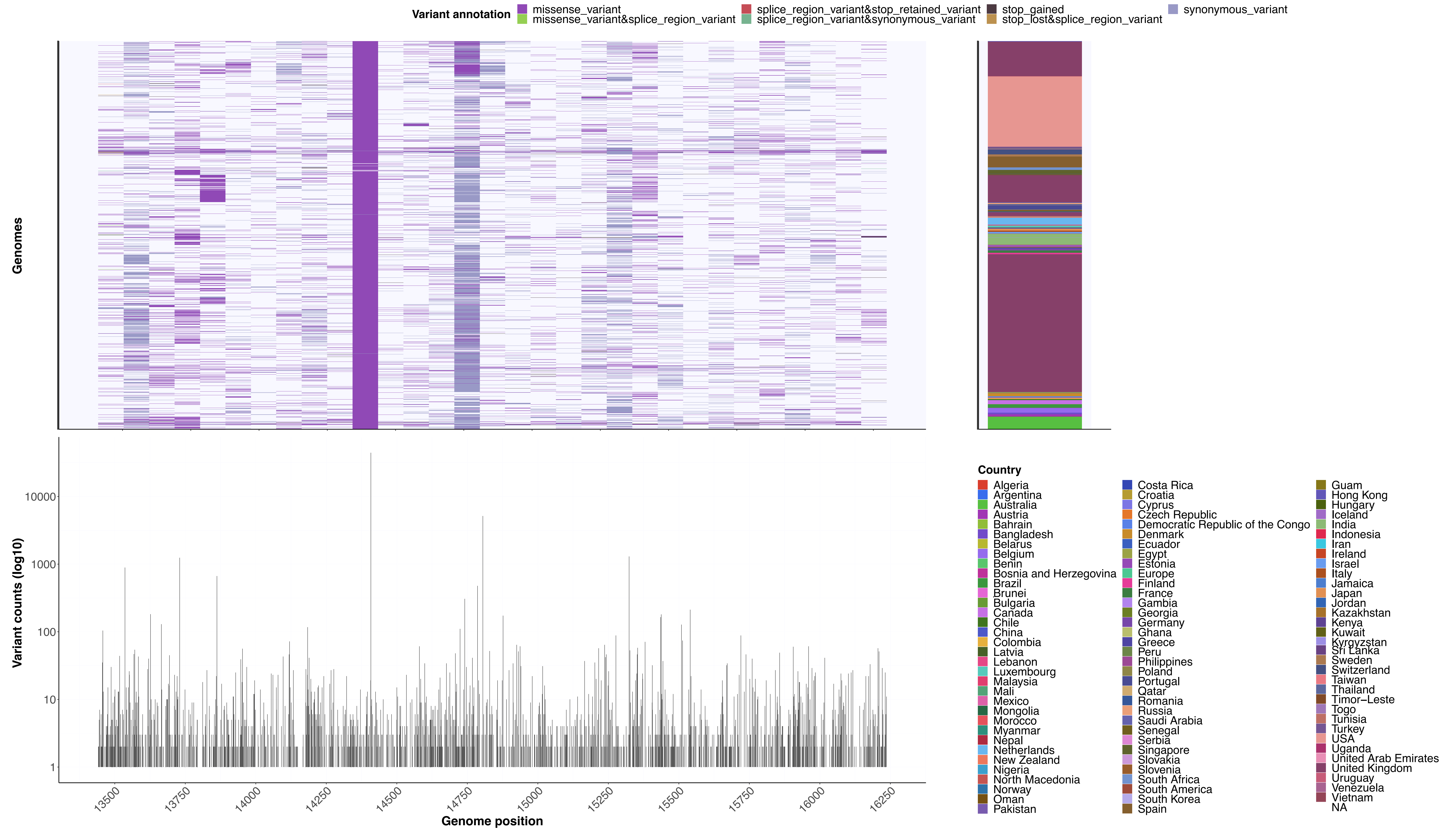

Supplement: Supplementary file 1 [file microorganisms-09-01094-s001.zip › Supplementary_files1/SI_Figure4.pdf]

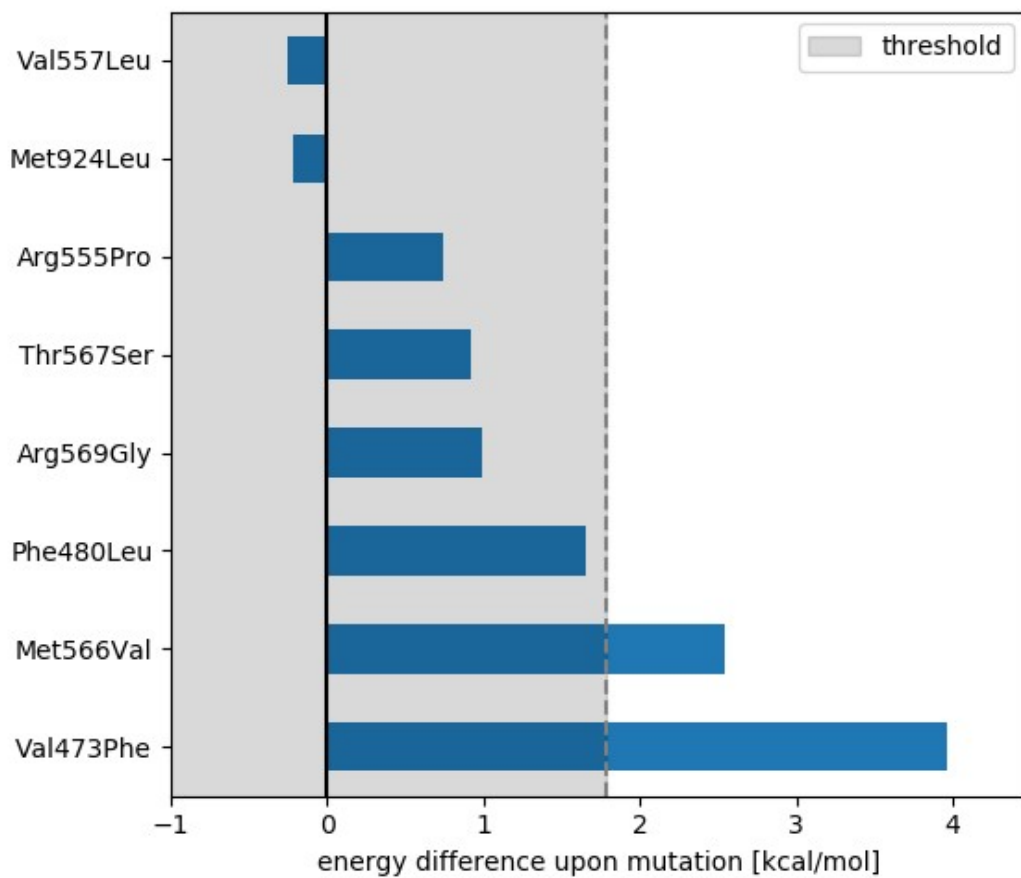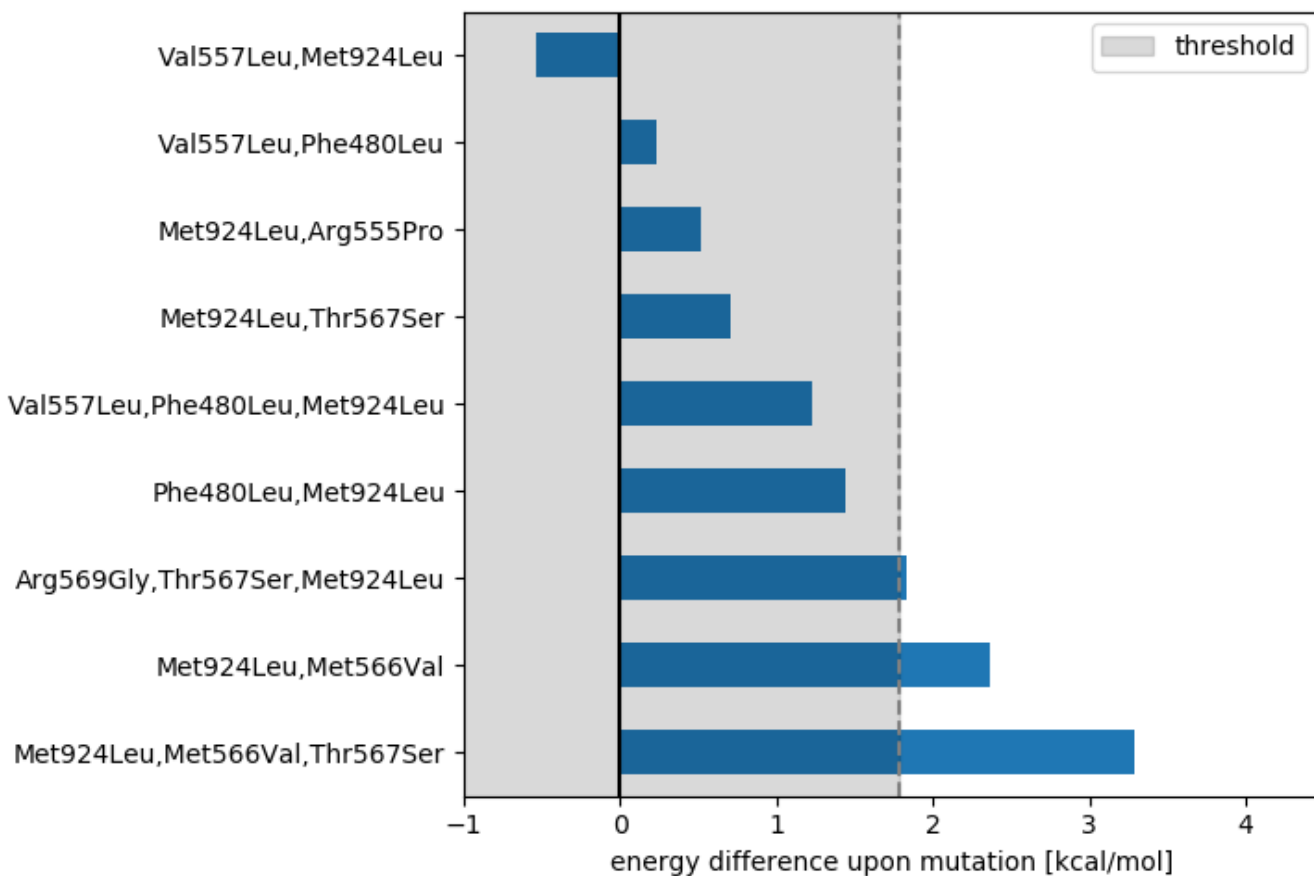

Supplement: Supplementary file 1 [file microorganisms-09-01094-s001.zip › Supplementary_files1/SI_Figure6.pdf]
